# CISD1 Is a Breast Cancer Prognostic Biomarker Associated with Diabetes Mellitus

**DOI:** 10.3390/biom13010037

**Published:** 2022-12-25

**Authors:** Fangfang Liu, Yifeng Dong, Fuyu Zhong, Haodan Guo, Pengzhi Dong

**Affiliations:** 1 Department of Breast Cancer Pathology and Research Laboratory, Tianjin Medical University Cancer Institute and Hospital, Tianjin 300060, China; 2 State Key Laboratory of Component-Based Chinese Medicine, Tianjin University of Traditional Chinese Medicine, 10 Poyanghu Road, Jinghai District, Tianjin 301617, China

**Keywords:** CISD1, breast cancer, diabetes mellitus, bioinformatics, ferroptosis

## Abstract

Women with diabetes mellitus are believed to have increased risk of developing breast cancer and lower life expectancies. This study aims to depict the association between the CISD1, the co-expressed genes, and diabetes mellitus to offer potential therapeutic targets for further mechanical research. The TCGA-BRCA RNAseq data is acquired. All the data and analyzed using R packages and web-based bioinformatics tools. CISD1 gene expression was evaluated between tumor bulk and adjacent tissue. Immune cell infiltration evaluation was performed. CISD1 expressed significantly higher in tumor tissue than that of the normal tissue, indicating poor overall survival rates. High expression level of CISD1 in tumor shows less pDC and NK cells penetration. There are 138 genes shared between CISD1 co-expressed gene pool in BRCA and diabetes mellitus related genes using “diabetes” as the term for text mining. These shared genes enrich in “cell cycle” and other pathways. MCODE analysis demonstrates that p53-independent G1/S DNA damage checkpoint, p53-independent DNA damage response, and ubiquitin mediated degradation of phosphorylated cdc25A are top-ranked than other terms. CISD1 and co-expressed genes, especially shared ones with diabetes mellitus, can be the focused genes considered when addressing clinical problems in breast cancer with a diabetes mellitus background.

## 1. Introduction

The most prevalent cancer among women worldwide is breast cancer, with the highest rates found in the most industrialized countries, such as Europe, North America, and Australia [1]. Several mechanisms that enable its progression, including evading apoptosis, dividing indefinitely, enhancing angiogenesis, resisting anti-growth signals, and producing its own growth signals. With a refined understanding of the molecular mechanism, there are many therapeutics in use and in clinical trials [2,3,4]. However, those patients with chronic conditions, such as diabetes, may face increased risk of suffering breast cancer or worse outcomes with breast cancer [5]. Mechanically, the development of breast cancer might be caused by dyslipidemia [6], hyperinsulinemia [7], and hyperglycemia induced by diabetes mellitus. Thus, metformin [8], thiazolidinediones [9] and other PPARγ agonists are thought have a potential for cancer prevention, although there are inconsistent results among studies [10,11,12].

Cancer cells accumulate iron and reactive oxygen species (ROS) to promote their metabolic activity and proliferation rate. In contrast, high levels of iron and ROS can also lead to increased oxidative stress and the activation of cell death pathways such as ferroptosis. As a result, different drugs that target iron and/or ROS metabolism have been proposed as anticancer medications. However, diabetes mellitus make it more complicated for the selection of the therapeutic treatments given the sustained hyperglycemia, hyperinsulinemia, insulin resistance (IR), and insulin-like growth factor-1 (IGF-1) play a role in cancer progression and promotion. Thus, personalized medicine and patient treatment based on precise molecular understanding is urgently needed.

Ferroptosis is an iron-dependent regulated form of cell death caused by accumulation of lipid-based ROS [13]. In addition to mitochondrial shrinkage and increased membrane density, mitochondrial cristae were reduced [14]. In the past few years, NEET proteins, a newly found iron-sulfur proteins (2Fe-2S), are pivotal players in iron regulation and ROS homeostasis. Specifically, CISD1 (CDGSH iron sulfur domain 1, CISD1—also known as mitoNEET) performs a key role in in promoting the proliferation of cancer cells and tumor growth [15]. It ensures maximal electron transport and oxidative phosphorylation capacity [16]. Knock-down CISD1 can significantly inhibit cancer cells proliferation and reduce uncontrolled reactive oxygen and iron accumulation [17]. Since it was reported that pioglitazone could stabilize CISD1, plenty of studies focused on the interaction between mitochondrial function and glutathione reductase metabolism [18,19,20]. Mutant CISD1 can lower glucose level in a model for studying human diabetes-related diseases [21]. A novel ligand for CISD1 named TT01001 improves diabetes [20]. All these achievements demonstrated that CISD1 can be a promising target for both breast cancer and diabetes mellitus cure. However, a more elaborated molecular mechanism is needed for clarifying the interplay between breast cancer, CISD1, and diabetes mellitus.

In this study, the RNA-seq data from Cancer Genome Atlas (TCGA) database was used to compare normal and tumor samples for CISD1 expression. Integrated bioinformatics methods were performed to examine the correlation between CISD1, co-expressed genes expression levels, and breast cancer clinical pathology features of breast cancer. Furthermore, diabetes mellitus related genes were added to identify the shared molecular pattern with CISD1 related co-expressed genes for future drug design or treatment options. This study aims to offer preliminary data for depicting the association between the CISD1, the co-expressed genes related, and diabetes mellitus to demonstrate the potential therapeutic targets for further mechanical research, even clinical practice when dealing with breast cancer especially those patients with diabetes mellitus.

## 2. Materials and Methods

### 2.1. Data Acquisition

BRCA and other tumor data from The Cancer Genome Atlas (TCGA) database (accessed on 20 December 2022, https://genome-cancer.ucsc.edu/) was used. Data was downloaded as level 3 HTSeq-fragments per kilobase per million (FPKM) before being converted to transcripts per million (TPM). All procedures in this study followed the Declaration of Helsinki.

### 2.2. Histological Verification of CISD1 Expression

**Figure 1:**
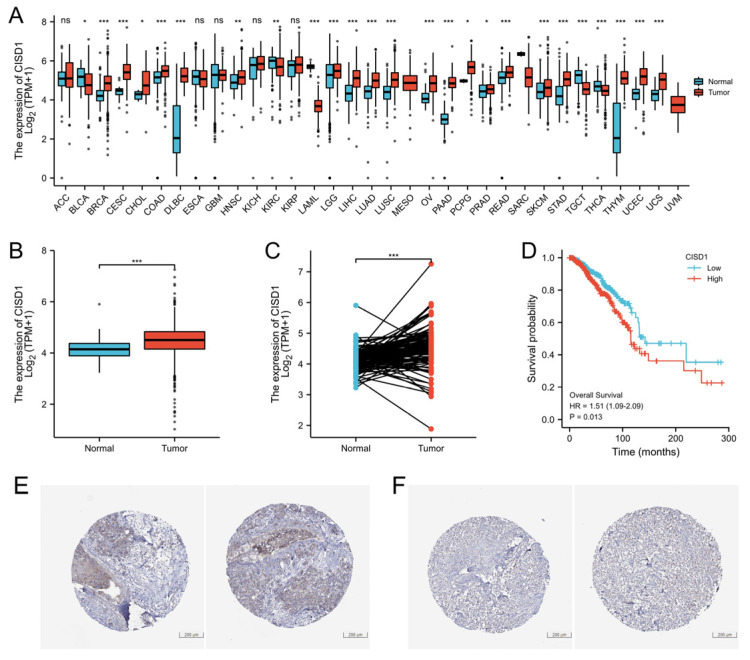
CISD1 expression correlated with life span. CISD1 expression varied in different cancers. (**A**) CISD1 expression level in non-paired; and (**B**) paired breast cancer samples; (**C**) Association between Overall Survival and CISD1 expression; (**D**) Verification of high-level; and (**E**) low-level; (**F**) expression of CISD1 in Human Protein Atlas. “ns”, “*”, “**”, and “***” represented no significance, *p*-values < 0.05, 0.01, and 0.001, respectively.

CISD1 expression was verified in Human Protein Atlas, a tissue-based map of the human proteome [22]. As shown in Figure 1E,F, CISD1 was stained, and the results were deposited in this database. Images representing high or low level CISD1 expression used in Figure 1E,F were obtained using the link, https://www.proteinatlas.org/ENSG00000122873-CISD1/pathology/breast+cancer#img. Accessed on 20 December 2022. Patients’ information was listed on the website. The Protein Atlas version was 21.1.

### 2.3. Correlation Analysis of CISD1 Expression and Clinical Features

For the correlation of the characteristics of patients and the CISD1 expression, a Venn Diagram was performed using R (v3.6.3) package ggplot 2 (v3.3.3).

### 2.4. Correlation Analysis of CISD1 Expression and Immune Infiltration

R package GSVA (v1.34.0) applying single-sample Gene Set Enrichment Analysis (ssGSEA) method was used to evaluate the association between the infiltration of immune cells and CISD1 gene expression [23,24].

### 2.5. Protein-Protein, Chemical-Protein Interaction Analysis

Protein–protein interaction was conducted in STRING v11.5 (accessed on 20 December 2022, https://string-db.org/). Chemical–protein interaction analysis was performed in STITCH v5.0 (accessed on 20 December 2022, http://stitch.embl.de/). Gene list of diabetes mellitus were obtained from Genecards, the Human Gene Database (accessed on 20 December 2022, https://www.genecards.org/) using “diabetes” as the term for collecting more genes related.

### 2.6. Gene Ontology (GO) Enrichment Analysis

Metascape (accessed on 20 December 2022, https://metascape.org) is a tool for gene annotation and pathway analysis [25]. In this study, shared genes list between the co-expression genes with CISD1 and diabetes mellitus were uploaded to Metascape, and enrichment analysis was performed. Additionally, transcription factors prediction, Molecular Complex Detection (MCODE) algorithm analysis were conducted by Metascape.

### 2.7. Transcription Regulating Prediction

Transcription regulation analysis was performed using The Gene Transcription Regulation Database (GTRD; accessed on 20 December 2022, http://gtrd.biouml.org/) [26].

### 2.8. Statistical Analysis

R (v.3.6.3) was used for all statistical analyses. *p* values less than 0.05 were considered statistically significant.

## 3. Results

### 3.1. High Expression Level of CISD1 in BRCA Tumor Correlates with Poor Survival Probability

In order to identify the difference of CISD1 expression between tumors and normal tissues, first, a pan-cancer analysis was performed to identify CISD1 expression level among 33 cancers data deposited in TCGA. As shown in Figure 1A, CISD1 protein contents were higher than normal tissues in 20 cancer types. CISD1 was highly expressed in a breast tumor, as shown in Figure 1B (non-paired samples). Further, comparison was conducted between breast tumor tissues and the adjacent tissues either. The result demonstrated that CISD1 was highly expressed in the breast tumor tissues, as shown in Figure 1C. Next, survival probability analysis was conducted using Kaplan-Meier analysis. Overall survival was calculated. The result indicated that there is a positive correlation between CISD1 expression and poor overall survival in patients with breast cancer (*p* = 0.013), as shown in Figure 1D. CISD1 expression level in tumor was verified by using Human Protein Atlas, and high and low expression levels of CISD1 are shown in Figure 1E,F, respectively.

### 3.2. Correlation between CISD1 Expression and Clinical Features

**Figure 2:**
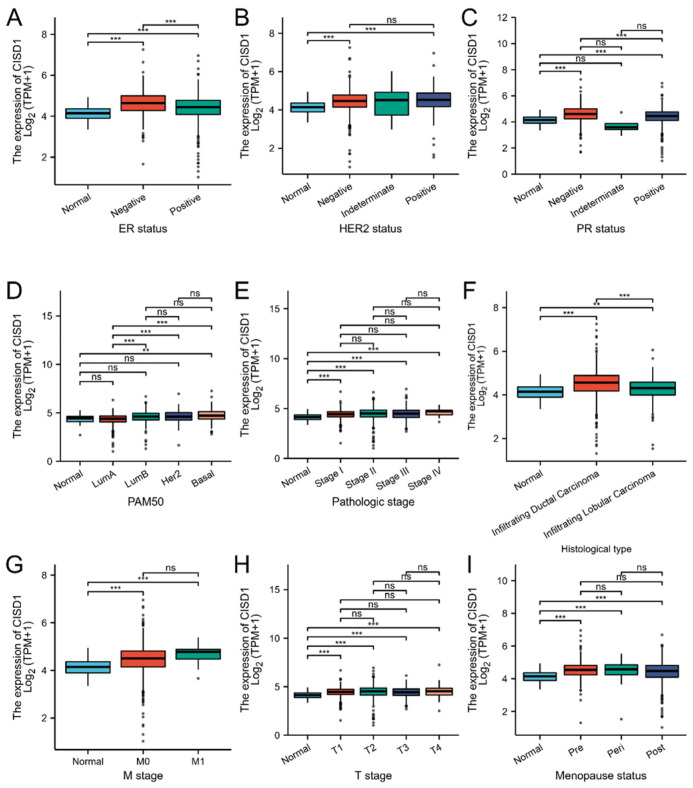
Correlation between the clinical features and CISD1 expression. Boxplots represented correlation between the CISD1 expression and ER status (**A**), HER2 status (**B**), PR status (**C**), PAM50 (**D**), pathologic stage (**E**), histological type (**F**), M stage (**G**), T stage (**H**), Menopause status (**I**). “ns”, “**”, and “***” represented no significance, *p*-values < 0.01, and 0.001, respectively.

Based on the mean value of CISD1 gene expression, the patients with breast cancer were divided into high expression level group (n = 542) and low expression level group (n = 541). The correlation between the clinical characteristics and CISD1 expression were evaluated and some associations were showed in Figure 2. CISD1 were significantly associated with many clinicopathologic parameters. N stage (*p* = 0.012), M stage (*p* = 0.047), Race (*p* < 0.001), Age (*p* = 0.022), Histological type (*p* < 0.001), PR status (*p* < 0.001), ER status (*p* < 0.001), and PAM50 (*p* < 0.001) were significantly connected with CISD1 expression. Detailed information is listed in Table 1.

### 3.3. The Correlation of CISD1 and Immune Cell Infiltration

**Figure 3:**
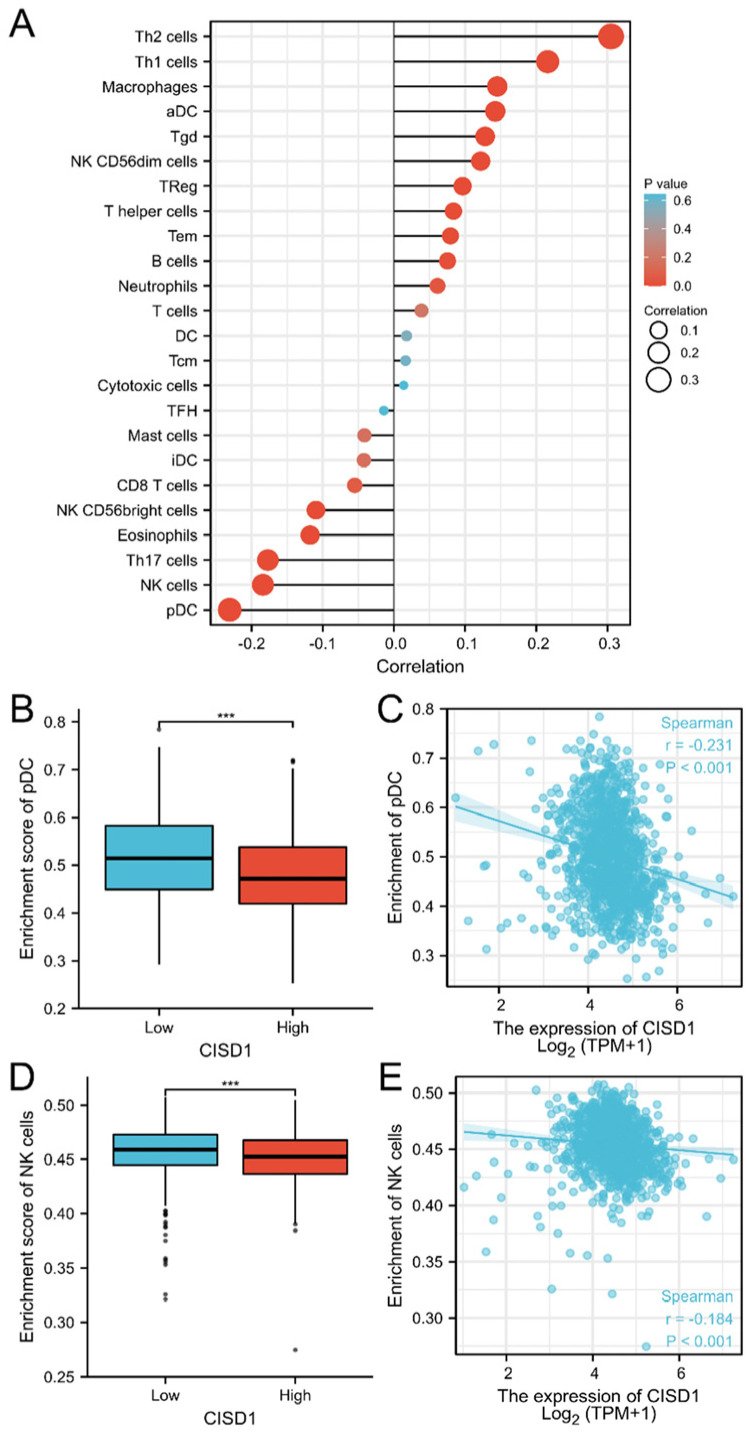
CISD1 expression associated with immune cell infiltration. Correlation between CISD1 expression inferred immune cells infiltration (**A**), pDC (**B**,**C**), and NK (**D**,**E**) negatively associated with CISD1 expression. “***” represented *p*-values < 0.001.

Next, the correlation between CISD1 expression and the immune infiltration was evaluated. As shown in Figure 3A, CISD1 expression was negatively associated with plasmacytoid dendritic cells (pDC) (*p* < 0.001), natural killer (NK) cells (*p* < 0.001), T helper 17 (Th 17) cells (*p* < 0.001), eosinophils (*p* < 0.001), NK CD56bright cells (*p* < 0.001), CD8 T cells (*p* = 0.067), interstitial dendritic cells (iDC) (*p* = 0.161), and mast cells (*p* = 0.168), while positively associated with T helper 2 (Th 2) cells (*p* < 0.001), T helper 1 (Th 1) cells (*p* < 0.001), macrophages (*p* < 0.001), activated dendritic cell (aDC) (*p* < 0.001), gammadelta T (Tgd) cells (*p* < 0.001), NK CD56dim cells (*p* < 0.001), regulatory T (Treg) cells (*p* = 0.001), T helper cells (*p* = 0.005), effector memory T (Tem) cells (*p* = 0.008), B cells (*p* = 0.012), neutrophils (*p* = 0.041), and T cells (*p* = 0.197). The pDC and NK cells enrichment were individually plotted (Figure 3B–E).

Detailed information had been listed in Table 2.

### 3.4. Co-Expression Genes, Protein-Protein Interaction, and Compound-Binding Analysis

**Figure 4:**
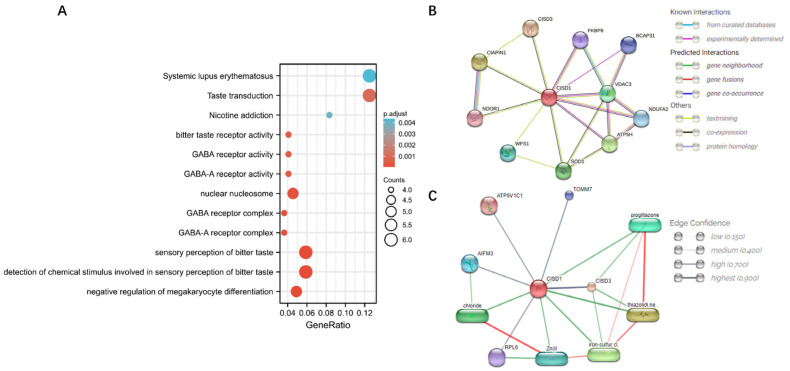
Co-expressed genes enrichment and interaction analysis. Co-expressed genes with CISD1 enrichment analysis (**A**), protein-protein interactions (**B**), and chemical-protein interactions (**C**) showed. Stronger associations represented by thicker lines. Chemical-protein interactions in green and interactions between chemicals in red.

As shown in Figure 4A, co-expressed genes with CISD1 were obtained and statistical analysis had shown that there were 717 genes (704 genes were positively correlated, while 13 genes were negatively correlated, |cor| > 0.3 and *p* < 0.05. cor = cor_spearman). Next, all these genes were conducted in an enrichment analysis, which the results showed that “Systemic lupus erythematosus” and “Taste transduction” ranked higher (*p* < 0.01) than other pathways. Next, first shell proteins interacted with CISD1 has been identified, as shown in Figure 4B. Among them, FKBP prolyl isomerase 8 (FKBP8), B cell receptor associated protein 31 (BCAP31), voltage dependent anion channel 3 (VDAC3), NADH ubiquinone oxidoreductase complex assembly factor 2 (NDUF2), D subunit of mitochondrial ATP synthase (ATP5H) had been experimentally testified that these genes interacted and co-expressed with CISD1 and might contribute a shared function. CDGSH iron sulfur domain 3 (CISD3), cytokine induced apoptosis inhibitor 1 (CIAPIN1), NADPH dependent diflavin oxidoreductase 1 (NDOR1), wolframin ER transmembrane glycoprotein (WFS1), and superoxide dismutase 1 (SOD1) were predicted functional partners with CISD1, which needs further improvement for the interaction between CISD1 and the genes mentioned above. Additionally, chemical-protein interaction was analyzed. The confidence results showed that pioglitazone, thiazolidi.ne, iron-sulfur cl., chloride, and Zn(II (equals to Zn (2+)) were predicted as functional partners with CISD1. Stronger associations were represented by thicker lines, as shown in Figure 4C.

### 3.5. Enrichment Analysis of Shared Genes between the Co-Expressed Genes with CISD1 and Diabetes Mellitus

**Figure 5:**
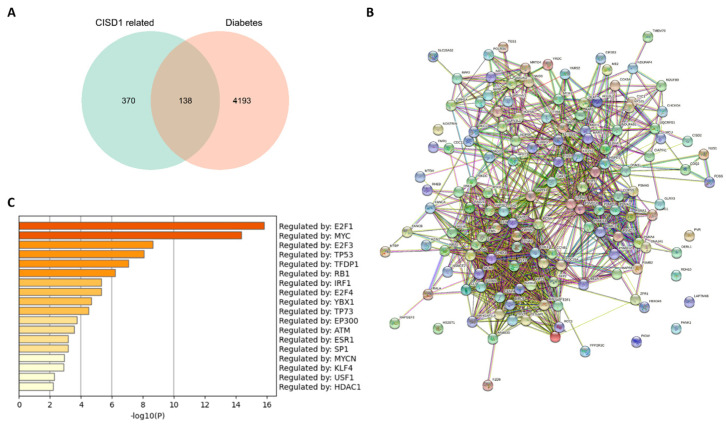
Shared genes between the co-expression genes with CISD1 and “diabetes mellitus”. A Venn diagram of shared genes between the co-expressed genes with CISD1 and “diabetes mellitus” (**A**); Protein-protein interaction network of the shared genes (**B**); and prediction of the transcription factors of the shared genes (**C**).

Given that pioglitazone was a drug used in the management of type 2 diabetes mellitus, which was also identified an interaction between pioglitazone and CISD1 in this study, we speculated that CISD1, a key role in ferroptosis, might be a significant target for treating BRCA with diabetes mellitus. Thus, it was necessary to present the correlation between the co-expressed genes with CISD1 in BRCA and diabetes mellitus. As shown in Figure 5A, there were 138 genes shared both in co-expressed genes with CISD1 and diabetes mellitus. Next, protein–protein interaction analysis was performed for these shared genes (Figure 5B). It was predicted that these genes were regulated by several pivotal transcription factors (Figure 5C).

Additionally, a transcription factor targets analysis was performed and 22 genes containing one or more binding sites for HSD17B8, as shown in Table 3, were found.

**Figure 6:**
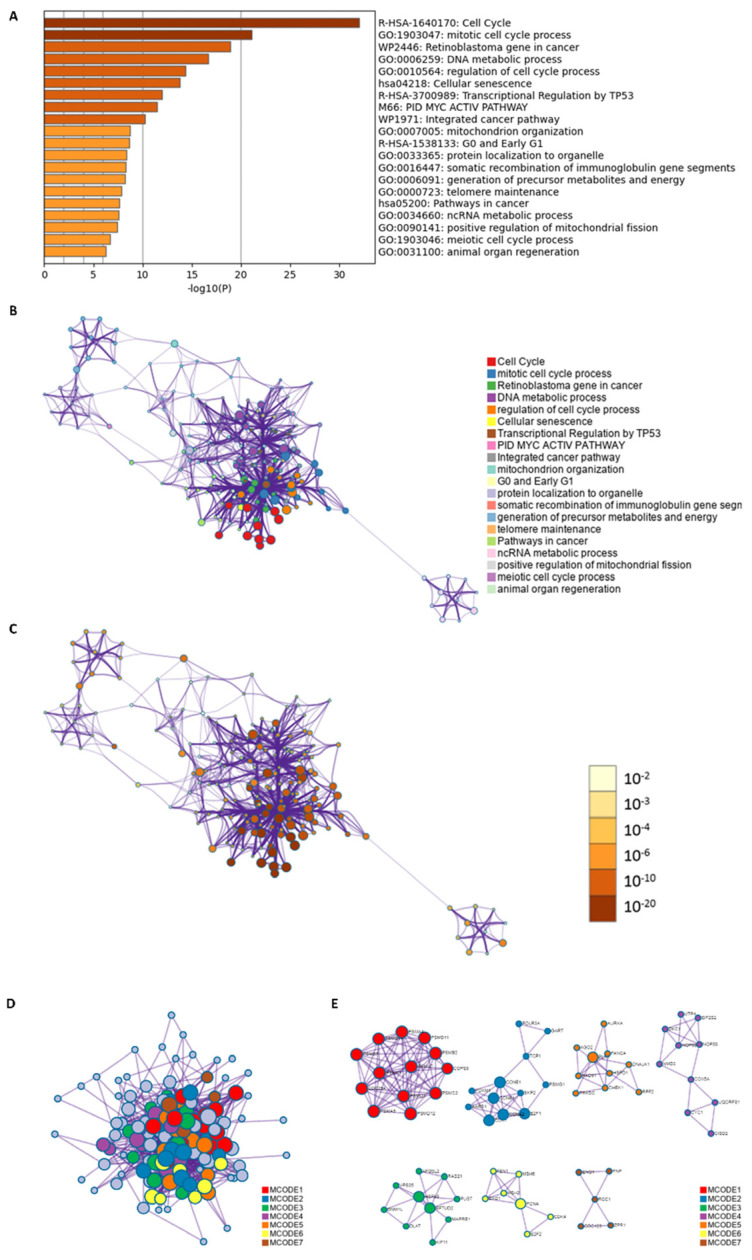
Gene enrichment analysis of the shared genes between genes co-expressed with CISD1 and “diabetes mellitus”. Pathways and functional terms enrichment results (**A**); Relationships of the terms listed by color (**B**); and *p*-value (**C**). Densely connected MCODE networks (**D**); and detailed MCODE networks (**E**).

These results suggested that certain cluster of genes might have similar mode of transcription regulation. Furthermore, gene enrichment was performed, and the results showed that cell cycle and mitotic cell cycle process ranked higher than other biological processes (Figure 6A). To further capturing the relationships between the terms, subsets of enriched terms had been selected and rendered as a network plot, in which terms with a similarity > 0.3 were connected. Each node represented an enriched term and was colored first by its cluster ID (Figure 6B), then by its *p* value (Figure 6C). For identifying densely connected networks, the MCODE analysis had been applied. Figure 6D showed the MCODE networks identified for the gene list.

Biological process and pathway enrichment analysis had been performed in MCODE component; top three scored terms were listed in Table 4. The results showed p53-Independent G1/S DNA damage checkpoint and other pathways might be the focus for further intervention.

## 4. Discussion

Breast cancer has become a serious threat to women’s health. It is the most common malignancy in women worldwide and is curable in 70–80% of cases if detected at an early stage and without metastatic spread. Breast cancer is heterogeneous at the molecular level, though there are different treatment strategies for different molecular subtypes. It is considered that targeting at iron and/or ROS metabolism can be used as a new strategy for developing anti-tumor drugs. However, due to the complex mechanism of ROS, most results are mixed. It is still necessary to find new targets that can regulate iron and ROS metabolisms, and perform an important role in tumor growth. In this work, an association between breast cancer and diabetes mellitus has been depicted through identifying the role of a ferroptosis related gene-CISD1.

The expression of CISD1in breast cancer and its correlation between the life span had been analyzed. CISD1 was positively associated with Overall Survival (*p* = 0.013). More importantly, detailed clinical information should be considered when grouping. Additionally, a correlation was found between CISD1 expression and clinical features. N stage (*p* = 0.012), M stage (*p* = 0.047), Race (*p* < 0.001), Age (*p* = 0.022), Histological type (*p* < 0.001), PR status (*p* < 0.001), ER status (*p* < 0.001), HER2 status, PR status, and PAM50 (*p* < 0.001) (Figure 2, Table 1) were significantly connected with CISD1 expression. These features are worthy to be concerned when making treatment plans for the patients. Next, CISD1 expression level were evaluated and varied in different cancer types (Figure 1A). It had been reported that loss of CISD1 could inhibit B-cell acute lymphoblastic leukemia cells proliferation [27]. Additionally, deficient CISD1 played a role in aged mice with heart failure [28]. Defected CISD1 mice showed a Parkinson’s Disease phenotype [29]. On the other hand, over expressed CISD1 could attenuate atherosclerosis [30], helped to improve hypoxic–ischemic injury [31]. These results showed that, CISD1 might play different role among cell types in tissues or organ systems. Thus, immune infiltration analysis could offer pivotal information for further interventions or mechanism studies. Our results showed that pDC, NK and Th17 and other cells were negatively correlated, while Th2, Th1 and macrophages and other cells were positively correlated with CISD1 expression in breast cancer (Figure 3). These results indicate that there might be a link between the high expression level of CISD1 and the inhibited the biological function of pDC and NK cells while might promote the Th2, Th1, and other cell physiological function.

As demonstrated above, CISD1 played significant roles in different types of symptoms or diseases, and its correlated networks must be varied according to the associated normal physiological or morbid pathological state. Therefore, co-expression genes of CISD1 in breast cancer were studied. Enrichment analysis results showed that these co-expressed genes with CISD1 contributed mostly to systemic lupus erythematosus, taste transduction, and other pathways, which in turn improved that CISD1 was related to immune system function from another perspective (Figure 4). Additionally, chemical-protein analysis had shown that a tested association between CISD1 and pioglitazone [21,32,33], which indicated that CISD1 might be a critical player both in breast cancer and diabetes mellitus. Thus, it was necessary to find out those shared genes between breast cancer and diabetes mellitus relating to CISD1 for further studies. As shown in Figure 5A, 138 shared genes were identified between the co-expressed genes with CISD1 and diabetes. Although there was complicated protein–protein interaction between these genes (Figure 5B), some of the shared 138 genes had similar trans-factors or regulatory mode (Figure 5C, Table 3). These shared genes were conducted gene enrichment analysis and the result showed that cell cycle and the mitotic cell cycle process obtained higher scores (Figure 6A). Targeting at cell cycle helped to inhibit cancer cell division [34]. In the progression of diabetes, changes in β cell mass and decreased beta cell proliferation or growth have been underappreciated. Changes in cell cycle progression enforce regulatory checks and balances on beta cells, similar to all other cells in our body [35]. Recently, there was a newly discovered mechanism that might be a key route for β cell proliferation regulation. In this work, amino acid availability and ROS levels were believed to be able to regulate postnatal β cell proliferation [36].

Although CISD1 had been indicated that it contributed to human breast cancer proliferation by inducing tumor growth, more detailed protein–protein, protein–nucleic acid interaction information was needed [17]. It had been reported that diabetes could be a risk factor for breast cancer [34]. Studies had demonstrated there were common pathways shared between diabetes and breast cancer [37]. Recently, studies targeting ferroptosis have identified a new strategy to treat breast cancer. Preliminary data supports that targeting CISD proteins could be considered as an effective way to improve hyperglycemia [38]. However, chronic diseases, such as diabetes, made it more complex to elaborate therapeutic protocols. Deeper understanding of the molecular mechanism correlated diabetes with breast cancer is imperative.

## 5. Conclusions

In summary, an integrated analysis was performed to demonstrate the possible link between breast cancer and diabetes via regulating CISD1 and related genes. This work provided a broader perspective to treat breast cancer and offered preliminary data for further research. However, more experimental evidence is needed to verify whether CISD1 could decrease pDC and NK cells or increase Th2, Th1, and macrophages infiltration. In line with this, the exact molecular mechanism for regulating CISD1 in different pathophysiological conditions could be completed.

## Figures and Tables

**Table 1:** Breast cancer clinicopathological characteristics and CISD1 expression.

Characteristic	Low Expression of CISD1	High Expression of CISD1	*p*
n	541	542	
**T stage, n (%)**			0.075
T1	148 (13.7%)	129 (11.9%)	
T2	296 (27.4%)	333 (30.8%)	
T3	80 (7.4%)	59 (5.5%)	
T4	16 (1.5%)	19 (1.8%)	
**N stage, n (%)**			0.012
N0	266 (25%)	248 (23.3%)	
N1	175 (16.4%)	183 (17.2%)	
N2	45 (4.2%)	71 (6.7%)	
N3	47 (4.4%)	29 (2.7%)	
**M stage, n (%)**			0.047
M0	451 (48.9%)	451 (48.9%)	
M1	5 (0.5%)	15 (1.6%)	
**Pathologic stage, n (%)**			0.099
Stage I	101 (9.5%)	80 (7.5%)	
Stage II	304 (28.7%)	315 (29.7%)	
Stage III	124 (11.7%)	118 (11.1%)	
Stage IV	5 (0.5%)	13 (1.2%)	
**Race, n (%)**			<0.001
Asian	23 (2.3%)	37 (3.7%)	
Black or African American	70 (7%)	111 (11.2%)	
White	405 (40.7%)	348 (35%)	
**Age, n (%)**			0.022
≤60	281 (25.9%)	320 (29.5%)	
>60	260 (24%)	222 (20.5%)	
**Histological type, n (%)**			<0.001
Infiltrating Ductal Carcinoma	346 (35.4%)	426 (43.6%)	
Infiltrating Lobular Carcinoma	139 (14.2%)	66 (6.8%)	
**PR status, n (%)**			<0.001
Negative	145 (14%)	197 (19.1%)	
Indeterminate	3 (0.3%)	1 (0.1%)	
Positive	371 (35.9%)	317 (30.7%)	
**ER status, n (%)**			<0.001
Negative	90 (8.7%)	150 (14.5%)	
Indeterminate	0 (0%)	2 (0.2%)	
Positive	430 (41.5%)	363 (35.1%)	
**HER2 status, n (%)**			0.647
Negative	290 (39.9%)	268 (36.9%)	
Indeterminate	6 (0.8%)	6 (0.8%)	
Positive	75 (10.3%)	82 (11.3%)	
**PAM50, n (%)**			<0.001
Normal	23 (2.1%)	17 (1.6%)	
LumA	338 (31.2%)	224 (20.7%)	
LumB	84 (7.8%)	120 (11.1%)	
Her2	33 (3%)	49 (4.5%)	
Basal	63 (5.8%)	132 (12.2%)	
**Menopause status, n (%)**			0.280
Pre	105 (10.8%)	124 (12.8%)	
Peri	19 (2%)	21 (2.2%)	
Post	364 (37.4%)	339 (34.9%)	
Age, median (IQR)	60 (50, 68)	57 (48, 66)	0.013

**Table 2:** CISD1 expression correlation with immune cells infiltration.

Cell Type	Correlation (Pearson)	*p* (Pearson)	Correlation (Spearman)	*p* (Spearman)
aDC	0.169	<0.001	0.142	<0.001
B cells	0.079	0.009	0.075	0.012
CD8 T cells	0.066	0.027	−0.055	0.067
Cytotoxic cells	0.062	0.039	0.014	0.646
DC	0.078	0.010	0.018	0.552
Eosinophils	−0.088	0.003	−0.118	<0.001
iDC	0.023	0.445	−0.042	0.161
Macrophages	0.175	<0.001	0.145	<0.001
Mast cells	−0.003	0.910	−0.041	0.168
Neutrophils	0.118	<0.001	0.061	0.041
NK CD56bright cells	−0.065	0.031	−0.109	<0.001
NK CD56dim cells	0.154	<0.001	0.122	<0.001
NK cells	−0.087	0.004	−0.184	<0.001
pDC	−0.201	<0.001	−0.231	<0.001
T cells	0.073	0.015	0.039	0.197
T helper cells	0.056	0.064	0.084	0.005
Tcm	−0.041	0.168	0.016	0.585
Tem	0.065	0.030	0.079	0.008
TFH	0.037	0.215	−0.014	0.639
Tgd	0.134	<0.001	0.128	<0.001
Th1 cells	0.240	<0.001	0.216	<0.001
Th17 cells	−0.141	<0.001	−0.177	<0.001
Th2 cells	0.282	<0.001	0.305	<0.001
TReg	0.111	<0.001	0.097	0.001

**Table 3:** Summary of enrichment analysis in transcription factor targets.

GO	Description	Count	%	Log10(P)	Log10(q)
M30019	HSD17B8 TARGET GENES	22	16.00	−12.00	−9.20
M19064	E2F Q6	11	8.00	−7.90	−5.10
M10115	E2F Q4	11	8.00	−7.90	−5.10
M29943	DLX6 TARGET GENES	16	12.00	−7.80	−5.00
M12497	E2F Q3	10	7.20	−7.00	−4.40
M5768	E2F1DP1RB 01	10	7.20	−6.90	−4.30
M9279	E2F1 Q6	10	7.20	−6.80	−4.20
M12402	E2F1DP1 01	10	7.20	−6.80	−4.20
M12555	E2F1DP2 01	10	7.20	−6.80	−4.20
M17867	E2F 02	10	7.20	−6.80	−4.20
M19298	E2F4DP2 01	10	7.20	−6.80	−4.20
M10526	E2F4DP1 01	10	7.20	−6.70	−4.20
M1485	E2F1 Q3	10	7.20	−6.70	−4.10
M8998	E2F1 Q4 01	9	6.50	−5.90	−3.40
M17117	E2F Q3 01	9	6.50	−5.80	−3.40
M3037	E2F1 Q6 01	9	6.50	−5.70	−3.30
M29995	HES4 TARGET GENES	13	9.40	−5.40	−3.00
M40770	ATXN7L3 TARGET GENES	9	6.50	−5.20	−2.80
M1905	SGCGSSAAA E2F1DP2 01	7	5.10	−4.90	−2.60
M102	E2F Q4 01	8	5.80	−4.80	−2.60

**Table 4:** Top 3 MCODE terms identified.

MCODE	GO	Description	Log10(P)
MCODE_1	R−HSA−69613	p53−Independent G1/S DNA damage checkpoint	−32.6
MCODE_1	R−HSA−69610	p53−Independent DNA Damage Response	−32.6
MCODE_1	R−HSA−69601	Ubiquitin Mediated Degradation of Phosphorylated Cdc25A	−32.6
MCODE_2	M176	PID FOXM1 PATHWAY	−14.5
MCODE_2	GO:0044772	mitotic cell cycle phase transition	−12.9
MCODE_2	GO:0044770	cell cycle phase transition	−12.7
MCODE_3	GO:0033365	protein localization to organelle	−5.8
MCODE_3	GO:0048285	organelle fission	−5.5
MCODE_3	GO:0000819	sister chromatid segregation	−4.9
MCODE_4	hsa03008	Ribosome biogenesis in eukaryotes	−9.9
MCODE_4	GO:0022613	ribonucleoprotein complex biogenesis	−8.5
MCODE_4	R−HSA−6790901	rRNA modification in the nucleus and cytosol	−8.5
MCODE_5	WP4016	DNA IR−damage and cellular response via ATR	−10.5
MCODE_5	WP4946	DNA repair pathways, full network	−7.3
MCODE_5	M258	PID BARD1 PATHWAY	−7
MCODE_6	R−HSA−5358565	Mismatch repair (MMR) directed by MSH2:MSH6 (MutSalpha)	−12
MCODE_6	R−HSA−5358508	Mismatch Repair	−11.9
MCODE_6	CORUM:286	PCNA−MSH2−MSH6 complex	−11.1

## Data Availability

Research data are stored in an institutional repository and will be shared upon reasonable request to the corresponding author Pengzhi Dong (pengzhiryn@gmail.com).
